# Glazing Affects the Fermentation Process of Sake Brewed in Pottery

**DOI:** 10.3390/foods13010121

**Published:** 2023-12-29

**Authors:** Koichi Tanabe, Honoka Hayashi, Natsuki Murakami, Yoko Yoshiyama, Jun Shima, Shinya Shoda

**Affiliations:** 1 Faculty of Agriculture, Ryukoku University, 1-5 Yokotani, Seta Oe-cho, Otsu 520-2194, Shiga, Japan; honopple7@gmail.com (H.H.); yyoshiyama@agr.ryukoku.ac.jp (Y.Y.); shima@agr.ryukoku.ac.jp (J.S.); 2 Research Center for Fermentation and Brewing, Ryukoku University, 1-5 Yokotani, Seta Oe-cho, Otsu 520-2194, Shiga, Japan; 3 Nara National Research Institute for Cultural Properties, 2-9-1 Nijo, Nara 630-8577, Japan; murakami-n33@nich.go.jp (N.M.); shinya.shoda@york.ac.uk (S.S.); 4 Department of Archaeology, BioArCh, University of York, York YO10 5DD, UK

**Keywords:** *Saccharomyces cerevisiae*, yeast, *Aspergillus flavus* var. *oryzae*, sake brewing, pottery, glazing

## Abstract

Sake (Japanese rice wine) was fermented in pottery for more than a millennium before wooden barrels were adopted to obtain a greater brewing capacity. Although a recently conducted analysis of sake brewed in pottery indicated that sake brewed in unglazed pottery contains more ethanol than that brewed in glazed pottery, little is known about the characteristics of sake brewed in pottery. In this study, we used two types of ceramic containers of identical size, one glazed and one unglazed, for small-scale sake brewing to evaluate the effects of glazing on fermentation properties. The following parameters were measured continuously in the sake samples over 3 weeks of fermentation: temperature, weight, ethanol concentration, and glucose concentration in sake mash. Taste-sensory values, minerals, and volatile components were also quantified in the final fermented sake mash. The results show that, in the unglazed containers, the temperature of the sake mash was lower and the weight loss was higher compared to the sake mash in the glazed containers. The quantity of ethanol and the levels of Na^+^, Fe^3+^, and Al^3+^ tended to be higher in the sake brewed in the unglazed pottery. A taste-sensory analysis revealed that umami and saltiness were also higher in the samples brewed in the unglazed pottery. These results suggest that glazing affects multiple fermentation parameters and the flavor of sake brewed in pottery. They may also suggest that the materials of the containers used in sake brewing generally affect the fermentation properties.

## 1. Introduction

Sake (Japanese rice wine) is a traditional alcohol that is brewed using rice, koji mold (*Aspergillus flavus* var. *oryzae*), and yeast (*Saccharomyces cerevisiae*). Koji mold secretes amylase for saccharification of rice starch, and the yeast converts glucose to ethanol during fermentation. In the traditional style of sake brewing, introduced more than 1300 years ago, the sake mash is squeezed in a cloth bag, and the clear flowthrough is collected as sake [1,2]. Sake is a complex beverage that contains a diverse array of compounds that contribute to its aroma, flavor, and overall sensory profile [3,4]. Volatile compounds such as ethyl acetate, n-propyl alcohol, isobutyl alcohol, and isoamyl alcohol, which characterize the flavor of sake, are produced during the fermentation process [5]. Ethyl caproate and isoamyl acetate, which are responsible for adding the apple and banana flavors to sake, respectively, are also produced during fermentation. The specific combinations and concentrations of these volatile compounds depend on various factors, including the sake-brewing process, rice choice, water quality, and yeast selection [6]. Over the centuries, various refinements have been introduced to the sake-brewing techniques and the containers used to ferment the sake mash. Sake was originally brewed using pottery as a container for the mash during fermentation. Approximately 400 years ago, at the start of the Edo Period, the increasing demand for sake led to the introduction of large wooden barrels for mass production. Today, contemporary sake brewers use enameled barrels, which enables stable microbial control of the sake mash. Although sake brewing had been performed with pottery for at least 800 years before the introduction of wooden barrels, little information has been accumulated about the characteristics of sake brewed in pottery.

In 2021, a cooperative effort was initiated by Yucho Shuzo, a sake brewery in Nara Prefecture, and the Nara National Research Institute for Cultural Properties to revive the style of sake production from the Nara period (around 1300 years ago). In this initiative, the brewers used two types of pottery, one glazed (produced in Shigaraki, Shiga prefecture) and the other unglazed (produced in Sabukaze, Okayama prefecture). Identical ingredients were used for fermentation in both containers. The sake brewed using unglazed pottery contained 16.8% (*v*/*v*) ethanol, whereas the ethanol concentration of the sake brewed using glazed pottery was slightly lower (15.4%). Sake brewed with unglazed pottery also had a higher amino acid content (2.8) than that brewed with glazed pottery (1.7) [7]. These results indicate that the decision of whether or not to use glaze affects the fermentation process and the flavor of sake brewed in pottery containers. However, no definitive conclusion can be reached because the size and form of the containers were significantly different: the volume of the glazed pottery container was 250 L, whereas that of the unglazed pottery container was 10 L. The size of the vessels that contain the sake mash can affect the physical parameters, including the uniformity of temperature, vaporization from the surface, and dissolved oxygen content, all of which are critical for the progress of fermentation [8,9].

In this study, we brewed sake on a small scale using two identically sized ceramic containers, one glazed and the other unglazed, to examine the effects of glaze on pottery-brewed sake. The temperature and weight change in the sake mash in the pottery containers were monitored during fermentation. The amounts of ethanol and glucose in the sake mash were also measured. Mineral measurement, volatile component analysis, and taste-sensory analysis were performed on the fermented samples to evaluate the flavor of the pottery-brewed sake. The information gathered about the role of glazing in determining the character of pottery-brewed sake will contribute to the development of modern sake-brewing techniques.

## 2. Materials and Methods

### 2.1. Saccharomyces cerevisiae Strains and Cultivation Media

The *Saccharomyces cerevisiae* strain, Kyokai No. 701, was obtained from the Brewing Society of Japan [10]. Yeast peptone dextrose (YPD) medium (1% yeast extract (Becton, Dickinson (BD) and Company, Sparks, MD, USA), 2% peptone (BD), and 2% glucose) was used for the cultivation of *S. cerevisiae*.

### 2.2. Ceramic Containers

The ceramic containers used in this study were produced by Kyoutou Yougyou Co. (Tajimi, Gifu prefecture, Japan). Both the glazed and unglazed containers were cylindrical and had the following dimensions: 9 cm diameter, 10 cm depth, and 4 mm thickness. The firing temperature (max. 1170 °C) was determined on the basis of a previous trial that considered the correspondence among pottery porosity, water absorption rate, and firing temperature.

### 2.3. Small-Scale Brewing of Sake

Small-scale brewing of sake was conducted in triplicate using two types of ceramic containers of identical size, one glazed and one unglazed, (i.e., three glazed and three unglazed ceramic containers were used). The following ingredients were added to each container: 105 g of 70%-milled pregelatinized rice (Tokushima Seikiku Co., Tokushima, Japan), 45 g of koji mold (Kojiya Kichiuemon, Shiga, Japan), 285 mL of tap water, and 0.5 mL of lactic acid (KENEI Pharmaceutical, Osaka, Japan). The ingredients were mixed, and the mixtures were kept at 15 °C for more than 3 h. The yeast cells (~1.4 × 10^8^) grown in YPD medium were then inoculated into each container, and the pottery containers were incubated in a temperature-controlled chamber at 15 °C [11]. Once a day, the ingredients in each container were thoroughly mixed using a spatula. Before mixing, the temperature of the ingredients (sake mash) at the bottom of the container was measured once a day using a stick-type temperature meter (TT-508, TANITA corporation, Tokyo, Japan). Aliquots (1 mL) of sake mash were collected on days 1, 3, 5, 7, 10, and 14 after inoculation. Aliquots were centrifuged at 12,000 rpm for 1 min (MX-307, TOMY SEIKO CO., LTD, Tokyo, Japan), and the supernatant of each sample was collected. The sake mash on day 21 after inoculation was centrifuged at 1700× *g* for 10 min (CR22N; Eppendorf Himac Technologies Co., Ibaraki, Japan), and the supernatant of each sample was collected. The continuously collected supernatants of sake mash were analyzed as sake samples using the methods described below.

### 2.4. Elemental Analysis Using iCAP

All samples were transferred into tubes and stored at −20 °C until processing. Samples were analyzed using an ICP-OES instrument (iCAP 7400 Duo; Thermo Scientific, Waltham, MA, USA) [12,13] with a concentric nebulizer and cyclonic spray chamber to determine the contents of micro- and macroelements. Data were expressed as the averages of three independent experiments and standard deviations.

### 2.5. Quantification of Glucose or Ethanol

The concentrations of glucose in the samples collected at days 1, 3, 5, 7, 10, 14, and 21 were determined using a Glucose Assay Kit-WST (DOJINDO Laboratories, Kumamoto, Japan) following the manufacturer’s instructions after proper dilution of the samples. The concentration of ethanol in the series of samples was determined using an F-kit for ethanol (R-Biopharm AG, Darmstadt, Germany) following the manufacturer’s instructions.

### 2.6. Analysis of Volatile Compounds

The representative volatile compounds in the sake samples were analyzed using a GCMS-QP2020 mass spectrometer (Shimadzu Corporation, Kyoto, Japan) and a DB-WAX-UI column (Agilent Technologies Japan, Tokyo, Japan) with a size of 30 m × 0.25 mm and a thickness of 0.25 μm. A 450 μL aliquot of each sake sample was used for the analysis, and 50 μL of 0.18% n-amyl alcohol (*m*/*z*: 55.00) was added to each sample as an internal standard. Each sample was placed in a 20 mL vial (TRAST HS vial, Shimadzu Corporation, Kyoto, Japan), and each vial was covered with a headspace cap, which was tightly crimped. The equilibrium was performed in an HS-20 headspace auto-sampler (Shimadzu Corporation, Kyoto, Japan). Each sample was injected as follows: incubation oven temperature of 70 °C, sample line temperature of 150 °C, transfer line temperature of 150 °C, without agitation, equilibrating time of 15 min, pressurizing time of 0.50 min, load time of 0.50 min, and injection time of 0.10 min. The carrier gas was helium that was injected at a flow rate of 1.23 mL/min. The oven temperature was held at 40 °C for 4 min, raised by 5 °C/min up to 100 °C, then raised to 200 °C at a rate of 40 °C/min, and held at 200 °C for 2 min (total time: 20.5 min). The interface temperature of the mass spectrometry detector (MSD) was 240 °C; the ion source temperature was 200 °C; and the electron impact energy was 70 eV. Selective ion monitoring (SIM) was conducted as follows: ethyl acetate (*m*/*z*: 61.00), n-propyl alcohol (*m*/*z*: 59.00), isobutyl alcohol (*m*/*z*: 43.00), isoamyl alcohol (*m*/*z*: 55.00), isoamyl acetate (*m*/*z*: 70.00), and ethyl caproate (*m*/*z*: 88.00).

### 2.7. Taste Analysis Using a Sensory Instrument

The taste of the sake samples brewed in pottery was quantitatively evaluated using a TS-5000Z taste-sensing system (Intelligent Sensor Technology, Kanagawa, Japan). A 75-mL aliquot of each sample was used for the analysis. The primary tastes analyzed were sourness (CA0), bitterness (C00), astringency (AE1), umami (AAE), and saltiness (CT0) [14]. The following aftertastes were also analyzed: bitterness (C00), astringency (AE1), and umami (AAE). A glazed pottery sample was used as a blank, and the relative voltages of the samples for each sensor were measured.

### 2.8. Statistical Analyses

The data obtained from three independent experiments were expressed as the mean ± standard deviation (SD). Differences in mean values between groups were tested using Welch’s *t*-test. A *p*-value of < 0.05 was considered statistically significant.

## 3. Results

### 3.1. Glazing on Pottery Affects the Weight and Temperature of the Mash during Sake Brewing

**Figure 1:**
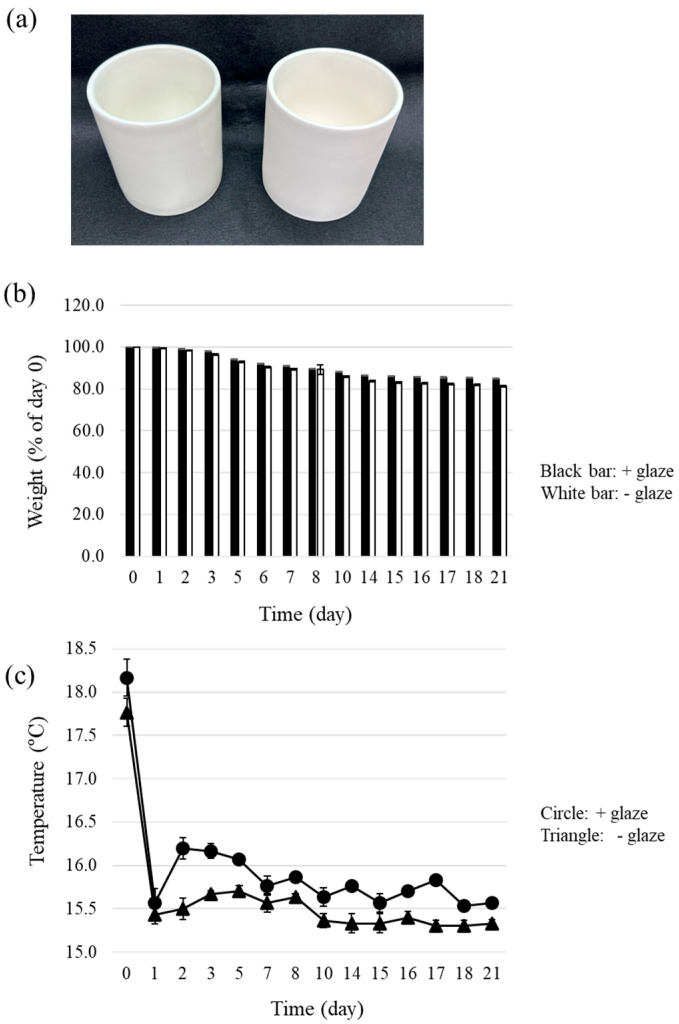
Weight and temperature changes during sake brewing in glazed and unglazed ceramic containers. (**a**) Appearance of the ceramic containers used in the experiments. Left: glazed, Right: unglazed. (**b**) Weight reductions in sake samples are represented as a percentage of the weight of the samples on day 0. Black bars, glazed; white bars, unglazed. (**c**) The temperature at the bottom of the containers was monitored. Circles, glazed; triangles, unglazed. Data represent the averages of three independent experiments; error bars indicate standard deviations.

We performed small-scale sake production with two identically sized ceramic containers, one glazed and one unglazed (Figure 1a). The environmental conditions (i.e., temperature, humidity, and barometric pressure) were identical for all fermentations. The weights of the sake mash and its containers were routinely measured during fermentation. The weight of each container and its sake mash continuously decreased during fermentation, with the average weight of the sake mash and containers in the glazed and unglazed containers declining to 84.9% and 81.3% of the initial weight, respectively (Figure 1b). The average temperature of the sake mash in the glazed containers was 0.2–0.7 °C higher than that in the unglazed containers throughout the measurement period (Figure 1c).

### 3.2. Sake Brewed in Unglazed Pottery Produced More Ethanol Than Sake Brewed in Glazed Pottery

**Figure 2:**
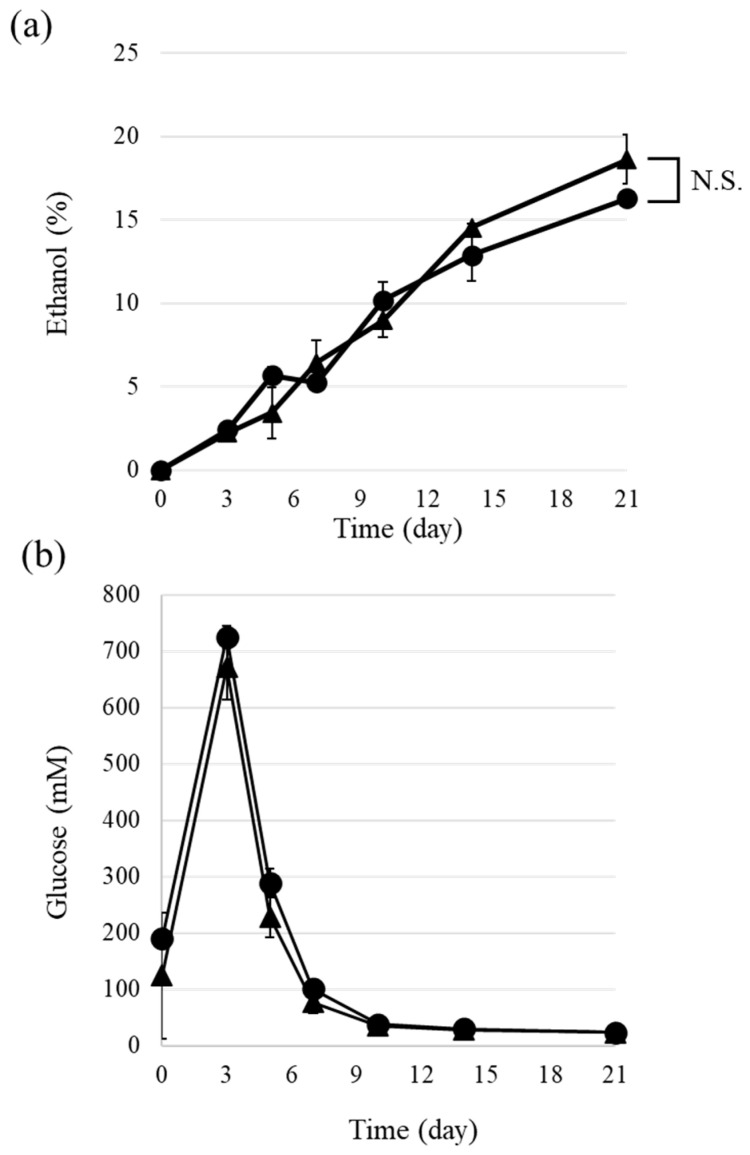
Concentrations of ethanol and glucose in the pottery-brewed sake samples. (**a**) The ethanol concentration (%, *v*/*v*) of aliquots of sake samples were determined. Circles, glazed; triangles, unglazed. N.S., not statistically significant (*p* > 0.05). (**b**) The glucose concentrations (mM) of aliquots of sake samples were determined. Circles, glazed; triangles, unglazed. Data represent the averages of three independent experiments; error bars indicate standard deviations.

The differences in weight and temperature of the sake mash indicated that ethanol fermentation proceeded differently in the glazed and unglazed containers. The ethanol and glucose concentrations of the periodically collected sake samples were measured (Figure 2). Until day 10, the average ethanol concentrations of the sake samples from the glazed and unglazed containers did not differ. On day 21, the average ethanol concentration of the sake samples from the unglazed containers appeared to be higher than that of the samples from the glazed containers, but the difference was not statistically significant (*p* = 0.0940) (Figure 2a). The average glucose concentration of the sake samples from the glazed containers was higher than that of the samples from the unglazed containers from day 1 to day 7, but this difference was also not significant (Figure 2b).

### 3.3. Sake Brewed in Unglazed Pottery Had More Saltiness and Umami Than That Brewed in Glazed Pottery

**Figure 3:**
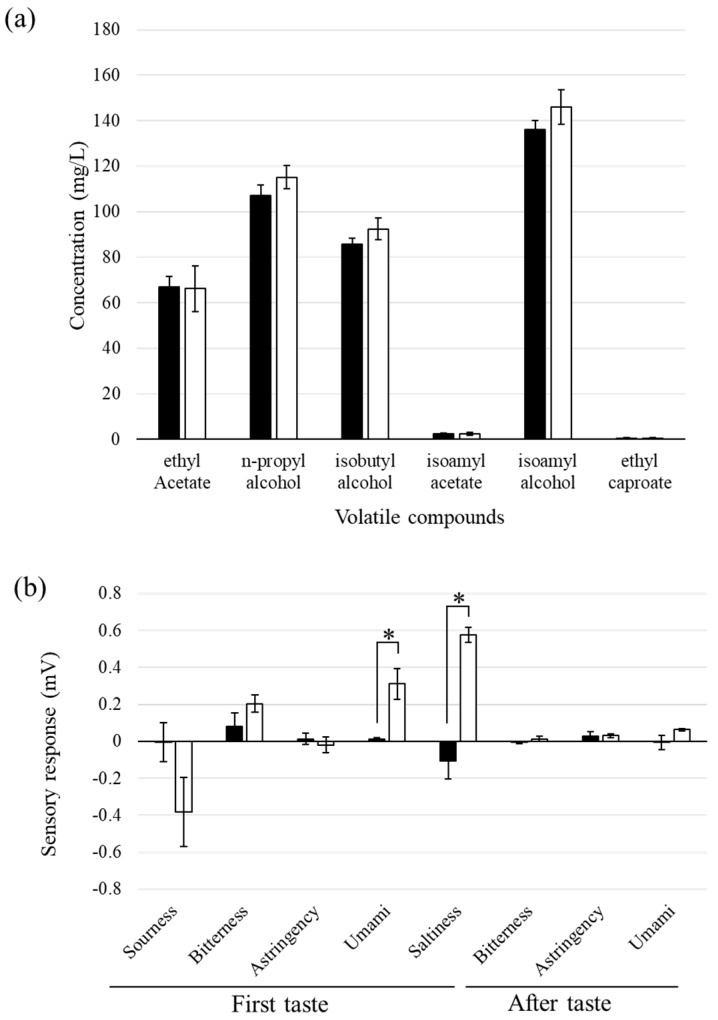
Taste and volatile components in sake samples brewed in glazed and unglazed ceramic containers. (**a**) Volatile compounds and representative compounds in sake were analyzed by using GC–MS. Black bars, glazed; white bars, unglazed. (**b**) The brewed sake samples were analyzed using a taste-sensory system. Asterisks indicate significant differences at *p* < 0.05. Data represent the averages of three independent experiments; error bars indicate standard deviations.

The sake mash sample from day 21 was centrifuged, and the supernatant was analyzed. The elemental analysis by using iCAP showed that higher concentrations of Na^+^, Fe^3+^, and Al^3+^ were detected in the sake from the unglazed containers than in that from the glazed containers (Table 1). The taste of sake was quantitatively analyzed using a taste-sensing system (Figure 3b). Among the primary tastes, the levels of umami and saltiness were significantly higher in the samples from the unglazed containers compared to those from the glazed containers, while the level of sourness was lower in the former. There was no clear difference in the aftertastes between the two types of samples. Volatile components are critical for the overall character of sake. The authentic aromatic components were analyzed by using Gas Chromatography–Mass Spectrometry (GC–MS) (Figure 3a). The average concentrations of n-propyl alcohol, isobutyl alcohol, and isoamyl alcohol were higher in the samples from the unglazed containers than those from the glazed containers, but the differences were not statistically significant.

## 4. Discussion

Recently, some sake breweries have started to use potteries for sake mash containers, in keeping with the ancient style, to produce sake with unique flavors or tastes. Although it is generally thought that glazing affects the ethanol fermentation of sake brewed in pottery, very little is known about the role of glazing in sake brewing. Here, we performed small-scale sake brewing with two identically sized ceramic containers, one glazed and the other unglazed, to examine the role of glaze in pottery-brewed sake.

Glaze is an important factor that affects the physical surface characteristics of pottery. Unglazed pottery allows the penetration of a small amount of water, whereas glazing almost completely blocks it. Water penetration may facilitate evaporation from the sides or bottoms of unglazed pottery. In our experiment, the average weight of the samples from the unglazed containers was lower than that of the samples from the glazed containers, with the respective weights finally declining to 84.9% (glazed) and 81.3% (unglazed) of the initial weights (Figure 1b). The loss of weight in sake mash in this experiment was thought to be caused by (i) the release of carbon dioxide accompanied by ethanol fermentation and/or (ii) the evaporation of water from the containers. Evaporation may also have reduced the temperature of the sake mash. The temperature of the sake mash in the unglazed pottery samples was consistently lower than that in the glazed pottery samples (Figure 1c). This result supports the idea that evaporation was more enhanced in the unglazed pottery.

The fluctuations in the glucose concentration of the sake mash during fermentation were quite similar between the two types of pottery (Figure 2b). The increase in glucose concentration reflects the saccharification of rice starch by amylase in the koji mold [1]. Because the optimal temperature for the activity of α-amylase is 50 °C [15], the enzymatic activity of α-amylase in *A. oryzae* was expected to be low under our experimental conditions (approximately 15 °C). The difference in temperature between the glazed and unglazed pottery fermentations did not result in significant differences in the amylase activities between the two types of pottery at temperatures much lower than the optimum temperature.

The ethanol concentrations in the unglazed pottery samples were higher than those in the glazed samples in the later stages of fermentation (Figure 2a). This trend in the ethanol concentration was also observed in the experiments conducted to confirm reproducibility, but the difference between the glazed and unglazed samples was not statistically significant. This result, together with the report from Yucho Shuzo, suggests that ethanol production by yeast was promoted in the unglazed pottery relative to the glazed pottery. Alcohol fermentation in *Saccharomyces cerevisiae* proceeds most efficiently at 25–35 °C, and a higher temperature is thought to be preferable for the accumulation of a high amount of ethanol under experimental conditions [16]. However, sake yeast strains exhibit high ethanol productivity (reaching 20%), efficient growth, and fermentation at low temperatures (below 15 °C) [10]. It is possible that the lower temperature in the unglazed pottery enhanced the ethanol fermentation, as the yeast strain K701 prefers lower temperatures for growth during the fermentation of sake [9,17]. It has also been reported that fermentation at low temperatures with this yeast strain results in a high concentration of ethanol in the final sake product [18,19]. These observations support the idea that the enhanced evaporation in the unglazed pottery caused a temperature drop and finally contributed to high ethanol fermentation.

The results of the GC–MS analysis reveal that there was no significant difference in the composition of the tested volatile compounds between the glazed and unglazed samples; however, the contents of n-propyl alcohol, isobutyl alcohol, and isoamyl alcohol were slightly higher in the unglazed pottery samples than in the glazed samples (Figure 3a). The biological pathway for the production of these alcohols has been intensively investigated because these compounds are thought to characterize the flavor of sake [5]. Yeast strains that produce high concentrations of these volatile compounds are required for developing sake with various flavors [20]. This result suggests that the container used for sake fermentation potentially contributes to the enhancement of these volatile compounds.

The taste-sensory analysis showed that the sake brewed in the unglazed pottery had more umami and saltiness than that brewed in the glazed pottery (Figure 3b). This result is consistent with the finding that greater amounts of amino acids were detected in the sake brewed in unglazed pottery compared to that brewed in glazed pottery at Yocho Shuzo brewery. The results of the elemental analysis via ICP reveal that 1.5 times more Na^+^ was detected in the unglazed pottery sample than in the glazed pottery sample (Table 1). It is possible that elements from the container elute during the long fermentation period in the absence of glazing. The difference in the concentration of Na^+^ was responsible for the enhanced saltiness of the unglazed pottery sample. Higher levels of Mg^2+^ and Ca^2+^ were detected in the glazed pottery samples than in the unglazed pottery samples (Table 1). It was expected that the yeast cells in the glazed pottery samples would grow better than those in the unglazed pottery samples because divalent cations in water, such as Mg^2+^ or Ca^2+^, are required for the growth of yeast cells during sake brewing [21]. Contrary to this prediction, a lower amount of ethanol was detected in the glazed pottery samples (Figure 2a). Slower growth of yeast cells leads to better yeast growth and more ethanol accumulation because slower growth at lower temperatures is ideal for ethanol production in the case of sake brewing.

We measured the amounts of ethanol, glucose, elements, and six volatile compounds in the sake-mash samples. The quantified compounds were selected as primary parameters for monitoring the fermentation of sake because (i) ethanol and glucose are the main products in sake produced by yeast and koji mold, respectively, and (ii) the six volatile compounds are thought to be the main compounds that characterize the flavor of sake. It should be considered that there are other compounds that also characterize the flavor sake, such as organic acids, amino acids, and volatile components other than those tested in this study [5]. These other compounds should be examined in future studies.

We found that glazing affected the fermentation and sensory parameters of pottery-brewed sake. Our results raise the possibility that the surface of the sake-mash container may affect the fermentation properties even in enameled barrels used in modern sake brewing. Factors directly related to fermentation in sake brewing, such as yeast strains, rice, water, or temperature control, have been investigated, whereas the influence of the container of sake mash has not been intensively investigated so far. This study will provide new insights into sake brewing by bridging ancient and modern techniques.

## 5. Conclusions

Sake was originally produced in pottery during the ancient period. It was suspected that glazing inhibited the evaporation of water from the container and had some effects on the fermentation properties of the sake. The fermentation properties of sake brewing were compared between sake brewed in glazed pottery and that brewed in unglazed pottery. The contents of ethanol, Fe^3+^, and Ca^2+^ in the sake brewed in the unglazed pottery tended to be higher than that in the glazed pottery. A taste-sensory analysis revealed that umami and saltiness were higher in the samples brewed in unglazed pottery. The material of the container used for sake brewing may therefore affect the fermentation properties.

## Figures and Tables

**Table 1:** Composition of elements in sake samples brewed in glazed and unglazed pottery.

	Concentration of Elements (mg/L)					
	+Glaze			−Glaze		
Na **	1.11	±	0.05	1.70	±	0.06
K	7.19	±	0.34	6.77	±	0.21
Ca **	3.12	±	0.04	2.56	±	0.02
Mg	1.66	±	0.07	1.55	±	0.06
Fe *	0.02	±	0.00	0.03	±	0.01
P	25.95	±	1.34	25.69	±	0.48
Al **	n. d.			0.08	±	0.01

Data represent the averages from three independent experiments ± standard deviations. * *p* < 0.05; ** *p* < 0.01; n.d., not detected.

## Data Availability

Data is contained within the article.
